# Speed Ratio in a Novel Multilayer Traffic Network for Urban Congestion Relief and Efficiency Gain

**DOI:** 10.3390/e28040469

**Published:** 2026-04-20

**Authors:** Wenna Liu, Bo Yang

**Affiliations:** 1 Data Science Research Center, Kunming University of Science and Technology, Kunming 650500, China; 2 Faculty of Science, Kunming University of Science and Technology, Kunming 650500, China; 3 Yunnan Key Laboratory of Complex Systems and Brain-Inspired Intelligence, Kunming University of Science and Technology, Kunming 650500, China

**Keywords:** multilayer traffic network, inter-layer speed ratio, congestion and efficiency

## Abstract

Based on observations of real-world transport systems such as bus-subway systems, street-motorway networks, and rail-air transport frameworks, in which high-speed layers are typically constructed above pre-existing low-speed networks to alleviate congestion and improve efficiency, this study proposes a method for constructing multilayer transport networks by strategically deploying the high-speed layer according to node betweenness centrality in the underlying low-speed network. The concept of speed ratio is introduced to quantify the speed difference within the multilayer network. The multilayer network is integrated into the following model: the user equilibrium flow assignment strategy model based on the Bureau of Public Roads function. Utilizing network efficiency, high-speed layer utilization ratio, and proportion of congested edges as metrics, we analyze the impact of: (1) inter-tier speed ratio, (2) low-speed layer topology, and (3) interlayer transfer costs on system performance. Key findings indicate: Under a given traffic demand, increasing the inter-layer speed ratio elevates network efficiency while shifting congestion from lower to upper layers; incorporation of long-range connections improves efficiency, alleviating traffic congestion; introducing interlayer travel speed may enhance efficiency in specific parameter regimes.

## 1. Introduction

Transportation networks are widely used in daily life. Studying transportation networks can optimize resource allocation, enhance transportation efficiency, alleviate congestion, and promote sustainable economic and social development. Extensive research has been conducted on various transportation networks, including bus networks [1,2], subway networks [3], airport networks [4], railway networks [5], urban road networks [6,7,8], and multi-layer coupled transportation networks [9,10,11]. These studies primarily focus on the topological properties, vulnerability and robustness of transportation networks, as well as the dynamic characteristics (such as congestion and network efficiency) on such networks.

Traffic models on networks primarily include: the traffic flow assignment strategy model based on node degree values The (ND-FAS model) [12,13,14,15,16], the user equilibrium flow assignment strategy model based on the Bureau of Public Roads function (BPR-UE model) [17,18,19,20,21,22,23,24,25]. ND-FAS model employs adaptive decentralized routing strategies that dynamically adjust path weights based on local topology metrics (e.g., node centrality, inter-layer coupling) to minimize congestion and maximize throughput across complex network structures. Yan et al. [12] proposed an effective path strategy based on node degree weight in a single-layer network, showing that network capacity increased by more than 10 times. The Gao et al. [13] extended this to a two-layer network and proposed a traffic-flow assignment strategy based on the coordinated regulation of macro and micro parameters, achieving a reasonable distribution of traffic between layers and maximizing network capacity. Ma et al. [14] found that the low-speed layer plays a dominant role in congestion based on a two-layer high-speed and low-speed network. On this basis, they constructed a three-layer network model and proposed a node degree resource allocation strategy that increased capacity by 1.5 times [15]. Further, through an edge addition strategy, they confirmed that the maximum capacity is achieved when all edges are added to the high-speed layer [16]. Ma et al. [11] analyzed the impact of routing strategies in a three-layer network on traffic and found that heterogeneous capacity allocation is more optimal. BPR-UE model is a traffic flow optimization approach that employs the BPR delay formula to estimate travel times and iteratively distributes flows until reaching a Wardrop equilibrium state where no driver can unilaterally reduce their travel time by changing routes. Wu et al. [17,18] compared congestion in topological networks based on the BPR function and found that scale-free networks have a significant advantage under high traffic volume. Sun et al. [19,20] combined community structure and indicated that related networks are more resistant to congestion. Xiao et al. [21] defined smoothness and found it to be linearly related to efficiency. Maniadakis et al. [22] combined the gravity model and confirmed that gravity networks are more resistant to congestion. He et al. [23] and Zhang et al. [24] respectively built models based on social signals and evacuation demands. Huang et al. [25] improved the BPR function and proposed a quantity adjustment model.

Empirical studies consistently reveal hierarchical or interconnected multilayer structures in real-world networks [26,27]. The multilayer architecture within transportation networks has also emerged as a pivotal focus in contemporary infrastructure research and can be divided into multilayer artificial networks and multilayer real-world networks. Morris et al. [28] generated the first-layer network using Delaunay triangulation on $N_{1}$ randomly distributed nodes within a unit circle, and then randomly selected $N_{2}$ points ($N_{2}\leq N_{1}$) from the first-layer network to generate the second-layer network using the same method. The overlapping nodes between the two layers were connected through a one-to-one coupling mode to establish inter-layer edges. Ding et al. [29] generated a street network in their paper and randomly assigned demographic attributes to each node. They then iteratively constructed the upper-layer rail network by sequentially selecting nodes in descending order of population size. Less ideally, the iteration process in this paper was capped at generating only 10 rail nodes. Tan et al. [30] and Li et al. [31] constructed a two-layer network based on two Barabási–Albert (BA) scale-free networks with the same number of nodes and average degree. They adopted three coupling strategies: assortative coupling, which establishes connections in descending order of node load; disassortative coupling, which establishes connections based on the descending order of node betweenness in the first-layer network and the ascending order of node betweenness in the second-layer network; and random coupling, which randomly selects nodes to establish connections. Sole-Ribalta et al. [32] established two two-layer network models: one based on the Erdős–Rényi (ER) random network model with the same number of nodes, and inter-layer edges were established sequentially according to node order; the other was generated based on the Random Geometric Graph (RGG) in two-dimensional space for short-range and long-range networks, with inter-layer edges established sequentially according to node order. Ma et al. [14] generated the low-speed layer and the high-speed layer based on the BA or ER model respectively, forming four combinations: ER on ER, ER on BA, BA on ER, and BA on BA. The number of nodes in the high-speed layer was half that of the low-speed layer. Half of the nodes in the low-speed layer were randomly selected to establish one-to-one coupling relationships with all the nodes in the high-speed layer, thus, forming inter-layer edges. Cheong et al. [33] generated the low-speed layer and the high-speed layer based on the BA or ER model, forming four combinations: ER on ER, ER on BA, BA on ER, and BA on BA. In the high-speed layer, the nodes are a subset of those in the low-speed layer. The overlapping nodes in the two layers establish inter-layer edges through one-to-one coupling connections. Tang et al. [34], based on Shanghai’s public transportation and metro station and line data, used the L-space method to establish independent sub-networks for public transportation and metro, constructing a two-layer network with stations as nodes and adjacent stations on the same line as edges. Inter-layer edges were established by identifying transfer stations within a 300-meter buffer zone of metro stations. Gu et al. [35] constructed a two-layer network based on the distribution of lines and stations from different transportation modes such as railways and aviation, with cities as nodes, and established inter-layer edges based on the transfer possibilities between different transportation modes in the same city. Du et al. [36] generated a three-layer aviation network consisting of a core layer, bridge layer, and edge layer based on the Chinese aviation network using the k-core decomposition algorithm, defining the inter-layer connections based on the actual connectivity between airports in the real flight network. In multilayer transport network studies, the interlayer speed ratio (ISR) has emerged as a critical metric for quantifying interlayer heterogeneity, driving recent advancements in multimodal system optimization [11,13,14,29,37,38].

In summary, most existing methods for constructing two-layer transportation networks rely on random connection strategies, which are inconsistent with real-world scenarios. Specifically, these conventional approaches either independently generate two single-layer networks and then establish interlayer connections via specific rules (e.g., random coupling) or first generate a lower-layer network and then randomly select nodes within it to form the upper layer. However, real-world two-layer transportation systems—such as bus-subway systems, street-motorway networks, and rail-air transport frameworks—follow a distinct construction logic, where the high-speed layer is built based on the structure of the pre-existing low-speed layer rather than random selection.

Notably, these real-world networks exhibit unique inherent characteristics that further highlight the limitations of existing methods. The low-speed layer, established earlier, features strong connectivity and numerous nodes/stops; in contrast, the high-speed layer—constructed later atop the low-speed layer to alleviate congestion, enhance efficiency, and reduce transport pressure—connects core areas with fewer nodes and significantly longer internodal distances. To align with this real-world structure, we select high-speed layer nodes based on betweenness centrality, an indicator that quantifies the proportion of shortest paths between all node pairs passing through a node, reflects its role as a critical transfer hub, and captures global structural importance (unlike degree centrality which only reflects local connectivity), thus, being more effective in identifying core nodes for congestion alleviation.

Motivated by the aforementioned inconsistencies between existing methods and real-world networks, as well as the unique characteristics of practical transport systems, this paper proposes a new method for constructing a two-layer transportation network upon an existing low-speed network, with node betweenness centrality as the core selection criterion. Additionally, considering that a key feature of these real-world networks is the significant speed difference between the high-speed and low-speed layers, this study focuses on discussing the impact of the speed ratio on network efficiency and congestion.

The organization of this paper is as follows. In Section 2, a high-speed and low-speed multilayer traffic network model is integrated into the user equilibrium flow assignment strategy model based on the Bureau of Public Roads function. In Section 3, the influence of factors such as model parameters, network scale, topology, and inter-layer costs in multi-layer networks on the performance of the traffic network model is analyzed and discussed. In Section 4, the research conclusions are summarized.

## 2. Model and Method

### 2.1. High-Speed and Low-Speed Multilayer Traffic Model

The considered multilayer network consists of two layers: a low-speed layer *L* with $N^{L}$ nodes and $M^{L}$ edges, and a high-speed layer *H* with $N^{H}$ nodes and $M^{H}$ edges. These two layers are interconnected via $M^{I}$ inter-layer links. In general, the construction cost of the high-speed layer is significantly higher than that of the low-speed layer, which leads to a smaller network scale (i.e., fewer nodes) in the high-speed layer. To improve operational efficiency, the physical distance between any two nodes in the high-speed layer is typically longer than that in the low-speed layer. For simplicity, we assume that the nodes in the high-speed network layer *H* are a subset of the nodes in the low-speed network layer *L*. We take a two-dimensional (2D) square lattice network to generate the low-speed layer *L*, and then use a subset of *L* to construct the high-speed layer *H*.

**Figure 1:**
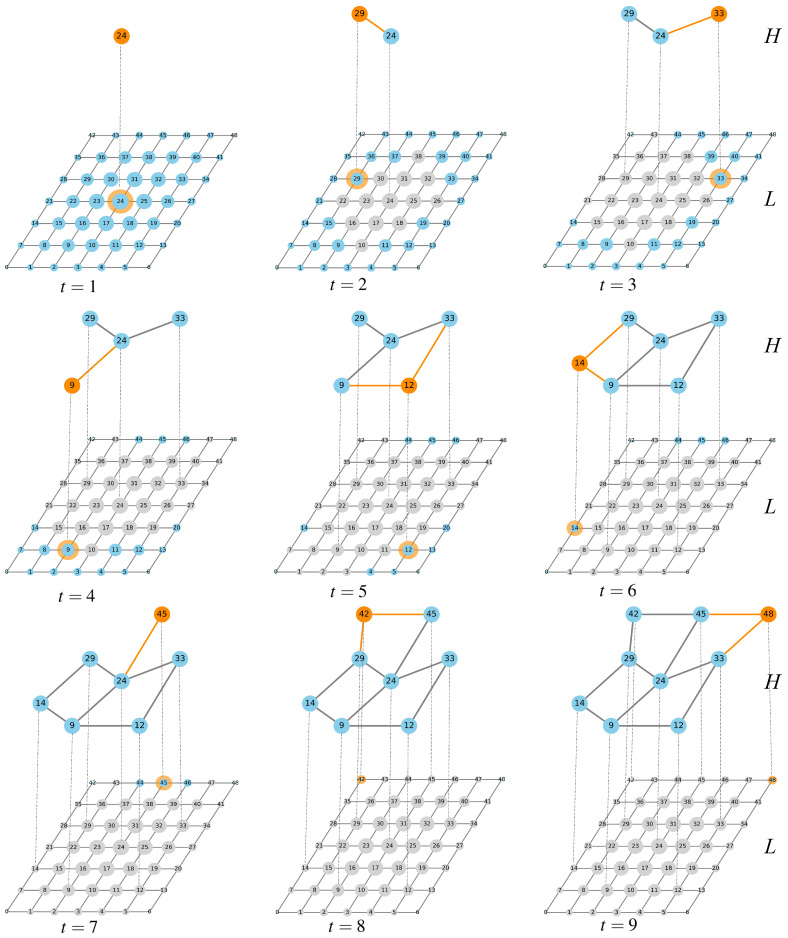
Multilayer network construction process where the nodes of high-speed network layer *H* form a subset of the nodes of low-speed network layer *L*, and nodes in common to both layers are considered to be coupled nodes (indicated as dashed lines), under the condition that layer *L* is a two-dimensional square lattice network with $N^{L}=49$, $N^{H}=9$ and τ=3. The larger the node, the greater the node betweenness value of that node. In the layer *L*, blue nodes represent the ones that may be selected, while gray nodes indicate those that cannot be chosen. In the layer *H*, orange nodes and orange edges denote the newly selected nodes and the newly formed edges each time.

Betweenness centrality effectively identifies nodes that serve as critical “bridges” in a network by quantifying the frequency with which a node appears on the shortest paths between other nodes. Such nodes may not necessarily have a high number of direct connections, but they play a significant role in controlling global connectivity and traffic distribution. In transportation network analysis, this metric can be used to accurately pinpoint key hubs that are prone to forming traffic bottlenecks. By specifically enhancing the capacity of these nodes or optimizing their path guidance, network congestion can be effectively alleviated, and overall system efficiency can be improved. Based on these characteristics, this study adopts betweenness centrality as the criterion for selecting nodes from the low-speed layer to form the high-speed layer. Considering that the high-speed layer typically comprises fewer nodes than the low-speed layer and that the spatial distance between high-speed layer nodes is generally larger, the algorithm skips a certain number of neighboring nodes after selecting a low-speed layer node to align with the structural characteristics of the high-speed layer. Based on this, we propose the following algorithm for constructing a dual-layer network comprising high-speed and low-speed layers. (1) Construct a low-speed layer network (e.g., two-dimensional square lattice, ER random network, BA scale-free network, etc.). Then, calculate the betweenness centrality for each node and store them in the candidate node set for the high-speed layer. (2) Select the node with the highest betweenness centrality from the candidate set and add it to the high-speed layer node set. Subsequently, remove all nodes from the candidate set whose topological distance to the selected node is less than τ. (3) Repeat step (2) until the high-speed layer node set reaches the predefined size or the candidate node set is empty. (4) For the high-speed layer node set, add high-speed layer edges between node pairs separated by a distance of exactly τ. Add inter-layer edges between node pairs with identical IDs in the low-speed and high-speed layers. The specific algorithm of high-speed network layer construction is presented in Algorithm 1. An illustration of the generation of a multilayer network is shown in Figure 1, with τ=3, $N^{L}=49$ and $N^{H}=9$.

In multilayer public transit networks (e.g., bus-subway systems), the operational velocity of each layer often differs significantly. The layer with higher operational velocity is termed the high-speed layer (e.g., subway lines), while the slower one is defined as the low-speed layer (e.g., bus routes). To quantify this inter-layer speed discrepancy, we introduce the preset speed ratio (γ) between the high-speed and low-speed layers, mathematically expressed as:

(1) $$\begin{array}{c} \gamma =\frac{v_{0}^{H}}{v_{0}^{L}}, \end{array}$$

where $v_{0}^{H}$ and $v_{0}^{L}$ are the free-flow speed of high-speed network layer *H* and the free-flow speed of low-speed network layer *L*, respectively. For simplicity, in computer simulations, $v_{0}^{L}$ is set to 1.
**Algorithm 1** High-speed layer construction algorithm**Input:**  $G^{L}\left(V^{L},E^{L}\right)$, distance threshold τ, number of high-speed network layer nodes $N^{H}$**Output:**  High-speed network layer graph $G^{H}\left(V^{H},E^{H}\right)$  1:  $V_{current}=V^{L}$, $V^{H}=\emptyset$, $E^{H}=\emptyset$  2:  **while** 
$|V^{H}|\;<N^{H}$
**do**  3:      $v_{max}=\arg\max\left(Betweenness\left(V_{current}\right)\right)$  4:      $V^{H}=V^{H}\cup \left\{v_{max}\right\}$  5:      $V_{remove}=\left\{v|v\in V_{current}\cap Distance\left(v_{max},v\right)<\tau \right\}$  6:      $E^{H}=E^{H}\cup \left\{\left(v_{max},u\right)|u\in V^{H}\cap Distance\left(v_{max},u\right)=\tau \right\}$  7:      $V_{current}=V_{current}-V_{remove}-\left\{v_{max}\right\}$  8:  **end while**  9:  **return** 
$G^{H}\left(V^{H},E^{H}\right)$10:  {The Betweenness() function returns the betweenness centrality value for each node within a set of nodes. The node with the maximum betweenness value is identified by the argmax() function, while the Distance() function calculates the distance between any two given nodes.}

### 2.2. The User Equilibrium Flow Assignment Strategy Model Based on the Bureau of Public Roads Function

To introduce dynamical congestion, each link (i,j) is assigned a random capacity $U_{ij}$ within the range [20,60], representing the maximum possible crossing flows on the link (i,j). For a link to operate normally, its traffic flow must meet the equation $Q_{ij}<\psi U_{ij}$ at all times; otherwise the link is regarded as congested, where $Q_{ij}$ denotes the traffic flow on the corresponding link, and total traffic flow $Q=\sum_{\begin{array}{c} i,j\in V,i\neq j \end{array}}Q_{ij}$. ψ is a tunable parameter with ψ≥1. In general, we assume ψ=1.5 in this paper [17].

To model congestion effects, the network loading mechanism differs from traditional weighted networks. If all travelers initially select the same path (typically the shortest in terms of travel cost), the link travel cost—which depends on link flow—increases as congestion develops. Consequently, the cost of this path may rise until it is no longer the minimum-cost option, prompting some travelers to switch to an alternative route. However, the alternative path may itself become congested, leading to further shifts. This iterative process, known as traffic assignment, continues until an equilibrium state is attained. When travelers consistently make rational route choices, the resulting flow pattern corresponds to a user-equilibrium (UE) assignment.

In traffic modeling, we treat the multilayer transportation network as a weighted network, where the weight of an edge represents the travel time required to traverse it. For any edge (i,j), the expected time $t_{ij}^{0}$ required to traverse it is calculated by the following formula:

(2) $$\begin{array}{c} t_{ij}^{0}=\frac{d_{ij}}{v_{0}^{F}}, \end{array}$$

where F∈{H,L,I}, denotes the layer to which edge (i,j) belongs, namely the high-speed layer, the low-speed layer, or the interlayer connection, respectively. $v_{0}^{F}$ denotes the free-flow speed. $d_{ij}$ is the distance of the edge (i,j). In computer simulations, the distance of any edge in artificial networks is set to 1, whereas in real-world networks, it is defined as the Euclidean distance between two nodes.

In this work, congestion effects can be described by the cost function. We mainly focus on the most widely used well-known formula:

(3) $$\begin{array}{c} t_{ij}=t_{ij}^{0}\left[1+\alpha {\left(\frac{Q_{ij}}{U_{ij}}\right)}^{\beta }\right], \end{array}$$

in urban traffic network proposed by US Bureau of Public Road, where α=0.15, β=4 for simplicity, $t_{ij}$ denotes the cost (weight) of the link (i,j). So the dynamic weights are described as:

(4) $$\begin{array}{c} t_{ij}=f\left(Q_{ij}^{UE}\right), \end{array}$$

where $Q_{ij}^{UE}$ is the volume of traffic in the link (i,j) under the UE condition, and f(.) is the cost function.

The solution algorithm can be stated as follows:

Step 1: Initialization. Perform an all-or-nothing assignment based on the free-flow travel time $t_{ij}^{1}=t_{ij}\left(0\right)$ for each link (i,j), obtain the initial link flows $\left\{Q_{ij}^{1}\right\}$. Set the iteration counter n=1.

Step 2: Update. For each link (i,j), set $t_{ij}^{n}=t_{ij}\left(Q_{ij}^{n}\right)$. That is, update the link travel times based on the current link flows.

Step 3: Direction finding. Perform an all-or-nothing assignment using the current link costs $\left\{t_{ij}^{n}\right\}$, obtain an auxiliary flow pattern $\left\{y_{ij}^{n}\right\}$.

Step 4: Line search. Find the optimal step size $\alpha_{n}$ by solving the following one-dimensional optimization problem:

(5) $$\begin{array}{c} \min_{0\leq \alpha \leq 1}\sum_{\left(i,j\right)}\int_{0}^{Q_{ij}^{n}+\alpha \left(y_{ij}^{n}-Q_{ij}^{n}\right)}t_{ij}\left(x\right)\;dx. \end{array}$$

Step 5: Move. For each link (i,j), update the link flows:

(6) $$\begin{array}{c} Q_{ij}^{n+1}=Q_{ij}^{n}+\alpha_{n}\left(y_{ij}^{n}-Q_{ij}^{n}\right). \end{array}$$

Step 6: Convergence check. If the convergence criterion is satisfied, e.g.,

(7) $$\begin{array}{c} \sqrt{\sum_{\left(i,j\right)}{\left(Q_{ij}^{n+1}-Q_{ij}^{n}\right)}^{2}}/\sum_{\left(i,j\right)}\left(Q_{ij}^{n}\right)\leq \varepsilon , \end{array}$$

(for some small convergence threshold ε>0 [39]), then stop; otherwise set n=n+1 and go to Step 2.

### 2.3. Related Indicators

For simplicity, in this paper, we assume that at each time step, unit traffic flow is generated between any two nodes of networks. Hence, the traffic flows are assigned by UE method, which assures the flow equilibrium in the network. The total volume of traffic is the sum of traffic flows in the network. We assume that even if the network is congested, the congested links are not removed from the network, but the costs on these links become infinite. Any link with flow exceeding its capacity and the total number *J* of congested links for different network topologies are calculated at each iteration with

(8) $$\begin{array}{c} M_{c}=\left\{\begin{array}{cc} M_{c}+1, & Q_{ij}>\psi U_{ij},\\ M_{c}, & \mathrm{otherwise}. \end{array}\right. \end{array}$$

So, the congestion factor *J* is defined as the percentage of links that are unavailable in the multilayer network:

(9) $$\begin{array}{c} J=\frac{M_{c}}{M}, \end{array}$$

where $M_{c}$ is the total number of congestion-critical edges in the multilayer network, and *M* is the total number of links in the multilayer network. When J=0, it indicates that there are no inaccessible links in the multilayer network, and J=1 indicates that the multilayer network is completely inaccessible. Similarly, the congestion factors of low-speed layer and high-speed layer are defined as $J^{L}=M_{c}^{L}/M^{L}$ and $J^{H}=M_{c}^{H}/M^{H}$, where $M_{c}^{L}$ ($M_{c}^{H}$) is the total number of congestion-critical edges in the low-speed (high-speed) layer, and $M^{L}$ ($M^{H}$) is the total number of links in the low-speed (high-speed) layer network.

The network efficiency *E* is formally defined as the summation of reciprocals of the shortest-path distances between all node pairs of low-speed network. This metric quantifies the global information propagation capacity of a network, where higher values indicate superior transmission performance. The mathematical formulation is expressed as:

(10) $$\begin{array}{c} E=\frac{1}{N^{L}\left(N^{L}-1\right)}\sum_{\begin{array}{c} i,j\in V^{L},i\neq j \end{array}}\frac{1}{D_{ij}}, \end{array}$$

where $N^{L}$ denotes the number of nodes in the low-speed layer. In this study, only the origin and destination of the shortest path are constrained to be low-speed-layer nodes, while high-speed-layer nodes are allowed to serve as transfer nodes. On the one hand, this setting arises from the fact that the high-speed-layer node set is a strategically selected subset of the low-speed-layer node set (i.e., $V^{H}\subset V^{L}$). On the other hand, it is also consistent with the characteristics of real multilayer traffic networks in which most trip origins and destinations are typically located in the low-speed layer. $D_{ij}$ denotes the shortest-path length between node *i* and node *j*, measured in travel time. This shortest path is searched over the multilayer traffic network composed of both the high-speed and low-speed layers, rather than being restricted to the low-speed layer alone. Since paths can switch between layers through interlayer connections, the high-speed layer can participate in the formation of shortest paths by providing candidate routes with higher travel speeds, thereby affecting the overall network efficiency. A larger *E* indicates a shorter average travel time between nodes and, thus, a higher network efficiency; conversely, a smaller *E* indicates a lower operational efficiency of the traffic system. Because a time-weighted network is adopted in this study, the shortest path is determined by the actual travel time, enabling the network efficiency to comprehensively reflect the effects of speed differences and congestion on traffic system performance.

The functional engagement of the high-speed network layer in facilitating connectivity between critical nodes of the low-speed layer can be quantified through a binary determination of whether the shortest-path routing between low-speed layer node pairs traverses high-speed layer edges. This topological cascade effect is formally captured by the high-speed layer utilization ratio *U*, defined as:

(11) $$\begin{array}{c} U=\frac{2n_{p}^{H}}{N^{L}\left(N^{L}-1\right)}, \end{array}$$

where $N^{L}$ represents the number of nodes in the low-speed network layer *L*; $n_{p}^{H}$ denotes the count of node pairs in the low-speed layer whose shortest paths traverse at least one edge from the high-speed layer.

## 3. Results and Discussions

This section systematically investigates the influence of factors such as model parameters, network scale, topology, and inter-layer costs in multi-layer networks on the performance of the traffic network model. First, on a two-dimensional square lattice network, the effects of the inter-layer speed ratio γ and traffic flows *Q* on efficiency, high-speed layer utilization, and congestion level are analyzed. Subsequently, the impact of network scale and the ratio of high-speed to low-speed layer nodes on performance is discussed. Further examination is conducted on how different network topologies and inter-layer transfer costs affect system performance.

### 3.1. The Influence of Inter-Tier Speed Ratio on Transportation in Two-Dimensional Square Lattice Network

In this section, the low-speed network layer is assumed to be a two-dimensional square lattice with $N^{L}$ nodes. The high-speed network layer containing $N^{H}$ nodes is constructed based on node betweenness. This study exclusively constrains shortest paths to those originating from and terminating at low-speed tier nodes, with high-speed tier nodes serving as intermediary transit points. This constraint is primarily because high-speed nodes constitute a strategic subset ($V^{H}\subset V^{L}$) selected from the low-speed node set wherein redundancy considerations are mathematically precluded. Moreover, the overwhelming majority of origin-destination pairs naturally reside within the low-speed tiers.

**Figure 2:**
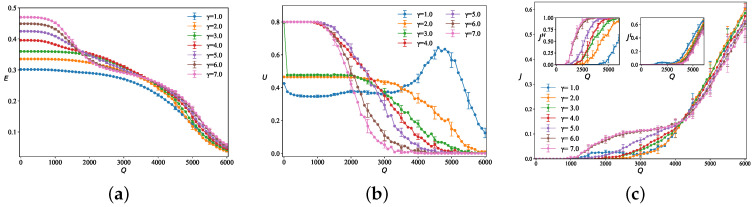
The curves depict (**a**) network efficiency *E*, (**b**) high-speed layer utilization ratio *U*, and (**c**) congestion factor *J* for different networks as functions of the traffic flow *Q* under different inter-tier speed ratios γ, with ε=0.01.

Fixing τ=3, $N^{L}=49$, $N^{H}=9$, $t_{ij}^{I}=0$, and ε=0.01, and averaging over 50 independent simulation runs, we investigate the impact of the inter-tier speed ratio γ and traffic flows *Q* on efficiency *E*, high-speed layer utilization *U* and multilayer network congestion factor *J*. Figure 2a shows that all curves start with a slow decline from Q=0, and larger values of γ correspond to higher initial values. Although the declining trends are relatively smooth, the efficiency curves for different γ tend to converge under high-flow conditions. Figure 2b illustrates that a larger γ results in higher utilization *U* under low-flow conditions, but lower *U* under high-flow conditions. At low loads, the high-speed layer is highly attractive; at high loads, however, its limited capacity causes traffic to be offloaded or rerouted to low-speed layers, leading to a sharp decline in utilization. The case of γ=1.0 is the only one where a peak in *U* is observed, indicating that when the speeds of different layers are identical, the utilization of the high-speed layer is optimized under high-flow conditions, while excessively high flow leads to a degradation in utilization. Figure 2c shows that as the traffic demand *Q* increases, the congestion factor of the low-speed layer $J^{L}$ exhibits a clear monotonic upward trend for all values of γ, with smaller γ corresponding to earlier and higher increases in $J^{L}$. The overall network congestion factor *J* remains close to zero under low demand, then rises with increasing *Q*, showing a generally monotonic increase. As for the congestion factor of the high-speed layer $J^{H}$, when γ is small, $J^{H}$ remains at a very low level throughout nearly the entire range. As γ increases, $J^{H}$ rises significantly and reaches relatively high values under medium to high demand, with the curve initially increasing rapidly and then tending to saturate. This indicates that larger γ causes traffic to concentrate on the high-speed layer earlier and more intensely, leading to earlier visible congestion in the high-speed layer.

**Figure 3:**
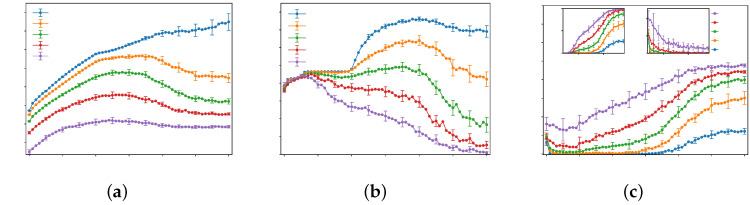
The curves depict (**a**) network efficiency *E*, (**b**) high-speed layer utilization ratio *U*, and (**c**) congestion factor *J* for different networks as functions of the inter-tier speed ratios γ under different traffic flow Q=1500,2000,2500,3000,3500.

**Figure A9:**
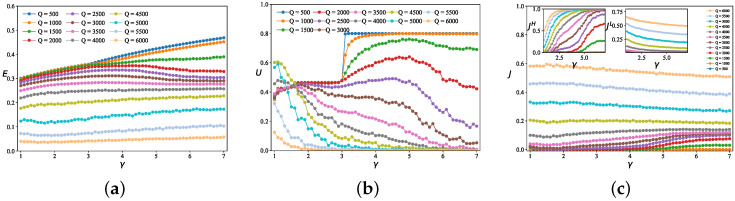
The curves depict (**a**) network efficiency *E*, (**b**) high-speed layer utilization ratio *U*, and (**c**) congestion factor *J* for different networks as functions of the inter-tier speed ratios γ under different traffic flow *Q*.

With the lower layer fixed as a two-dimensional square lattice ($N^{L}=49$, $N^{H}=9$) and parameters τ=3, $t_{ij}^{I}=0$ and ε=0.01, Figure 3 illustrates the system behavior as the inter-tier speed ratio γ increases under different levels of total traffic demand *Q*. The overall efficiency *E* decreases consistently with growing *Q*. Utilization *U* rises under low demand, exhibits a peak-and-decline pattern under moderate demand, and trends downward under high demand. As γ increases, congestion in the high-speed layer becomes more severe, while congestion in the low-speed layer is alleviated. The complete parameter diagram is shown in Figure A9 in the Appendix B.2.

As shown in the figure, when the network capacity is between 2000 and 3500, the network efficiency exhibits a unimodal characteristic, that is, there exists an optimal speed ratio that maximizes the network efficiency, which is approximately around γ = 3.5. Meanwhile, a reasonable speed ratio can alleviate congestion. The possible reasons for these phenomena are as follows: When the total traffic volume in the network is small, the network efficiency increases monotonically with the increase of the speed ratio. When the total traffic volume reaches a certain value, congestion begins to occur in the network; if there is no speed difference between layers, the upper-layer edges introduced in the network only have an impact on a small number of routes. As the speed ratio gradually increases, more and more OD pairs near the origin and destination choose the high-speed layer, which further greatly improves network efficiency and reduces congestion in the network. When the traffic volume in the network is large, the entire network is in an overloaded state, and the impact of the speed ratio on network efficiency and congestion becomes less obvious.

### 3.2. The Influence of Network Scale on Multilayer Traffic Model

This section examines the impact of network scale on the performance of the traffic model. Since the high-speed layer nodes are selected from the low-speed layer based on node betweenness and are typically fewer in number, we analyze the effect of network scale from the perspective: gradually increasing the number of high-speed layer nodes while keeping the number of low-speed layer nodes fixed.

**Figure 4:**
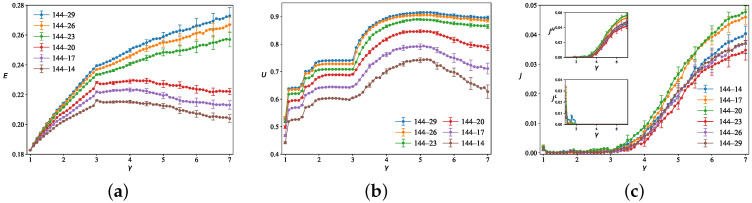
The impact of different numbers of high-speed layer nodes on traffic dynamics is presented for (**a**) network efficiency *E*, (**b**) high-speed layer utilization *U*, and (**c**) congestion factor *J*, as a function of the inter-tier speed ratio γ. Parameters are fixed at $N^{L}=144$ with $N^{H}/N^{L}$ ratios ranging from 0.1 to 0.2.

To investigate the impact of high-speed layer node density on network performance, we maintain a fixed number of low-speed layer nodes $N^{L}=144$ while systematically increasing the number of high-speed layer nodes $N^{H}$. The $N^{H}/N^{L}$ ratios are approximately 0.1, 0.12, 0.14, 0.16, 0.18, 0.2, corresponding to $N^{H}$ values of 14, 17, 20, 23, 26, and 29, respectively. Other parameters are fixed τ=3, $t_{ij}^{I}=0$, ε=0.01, and Q=3500, and averaging over 50 independent simulation runs. As shown in Figure 4a, when the number of upper-layer nodes is relatively large, *E* exhibits a sustained monotonic increase with γ, and a larger number of upper-layer nodes leads to a greater improvement in *E* and a higher final value. However, when the number of upper-layer nodes decreases to a certain level, *E* initially rises rapidly with increasing γ, but begins to decline significantly after reaching a critical threshold. This indicates that when the number of high-speed layer nodes is too small, even a further increase in γ is insufficient to continuously improve the overall efficiency. Figure 4b shows that as the inter-layer speed ratio γ increases from 1 to 7, the overall trend of the high-speed layer utilization *U* exhibits an initial rapid increase followed by a slight decline. For a given γ, *U* increases more slowly as the number of upper-layer nodes grows. *U* reaches its maximum around γ≈5, after which it gradually decreases with further increases in the inter-layer speed ratio γ. Figure 4c shows that the overall network congestion *J* and the high-speed layer congestion $J^{H}$ remain at very low levels when γ≤3, but increase significantly from γ≈3 onward. The low-speed layer congestion $J^{L}$ is generally high at γ≈1, yet it drops sharply to near zero when γ increases slightly to around 2. Under the current traffic demand, the optimal operating range of γ in which congestion is minimized lies approximately between 2.0 and 3.0.

### 3.3. The Influence of Network Structure on Multilayer Traffic Model

**Figure 5:**
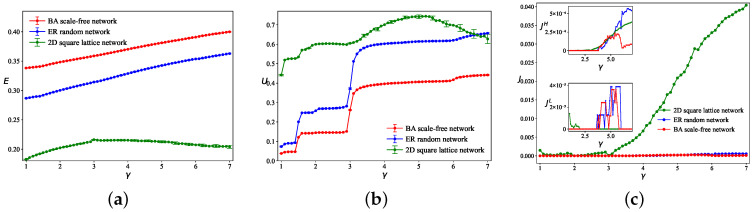
Given Q=3500, the curves depict (**a**) network efficiency *E*, (**b**) high-speed layer utilization ratio *U*, and (**c**) congestion factor *J* for different networks as functions of the inter-tier speed ratio γ.

While prior studies have adopted the two-dimensional square lattice as the canonical low-tier network topology, a critical scientific inquiry emerges: how do alternative network architectures modulate the multilayer efficiency-congestion duality? We, therefore, propose a comparative network morphogenesis framework by constructing two prototypical networks—Erdos–Rényi (ER) random networks (exhibiting Poissonian degree distribution) and Barabási–Albert (BA) scale-free networks (manifesting power-law connectivity)—while maintaining identical node counts and comparable edge densities to the baseline lattice. The following results are averaged over 50 trials with fixed parameters τ=3, $t_{ij}^{I}=0$, ε=0.01, and Q=3500. The low-speed layer for both the ER random network and the two-dimensional square lattice contains $N^{L}=144$ nodes and $M^{L}=264$ edges, while the BA scale-free network has $N^{L}=144$ and $M^{L}=284$. All three high-speed layers consist of $N^{H}=14$ nodes. Figure 5 plots the network efficiency *E*, high-speed layer utilization ratio *U*, and congestion factor *J* against the inter-tier speed ratio γ for these topologies. Comparative analysis reveals that the two-dimensional square lattice performs poorly, with the lowest *E* and the highest *J*, indicating greatest congestion vulnerability. The BA scale-free network achieves the highest *E* and the lowest *J*, demonstrating superior congestion resistance. The *J* of the two-dimensional square lattice network increases sharply with γ, making it the most congested topology, while the *J* of the BA and ER networks remain at very low levels throughout.

### 3.4. The Influence of Interlayer Transfer Costs on Multilayer Traffic Model

**Figure 6:**
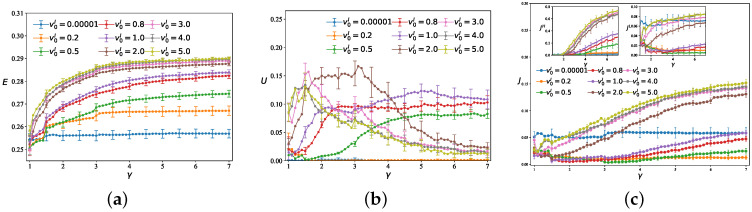
Given Q=3500, the plots depict: (**a**) network efficiency *E*, (**b**) high-speed layer utilization ratio *U*, and (**c**) congestion factor *J*, as a function of the inter-tier speed ratio γ under different inter-layer free-flow speeds $v_{0}^{I}$.

Prior studies fundamentally neglect inter-layer transition costs by assigning zero weight to inter-tier connections $t_{ij}^{I}=0$. This section pioneers the investigation into how graded inter-tier velocities—quantified through the generalized weight function $t_{ij}^{I}=d_{ij}/v_{ij}^{I}$—governs the efficiency and congestion in multilayer networks. We consider a two-dimensional square lattice with fixed parameters τ=3, $N^{L}=49$, $N^{H}=9$, ε=0.01, and Q=3500, and averaging over 50 independent simulation runs. Figure 6 plots the network efficiency *E*, high-speed layer utilization ratio *U*, and congestion factor *J* against the inter-tier speed ratio γ for different inter-layer velocities $v_{0}^{I}$. The results show that *E* generally increases with γ. The inter-layer velocity exerts a significant positive impact on *E*: a higher $v_{0}^{I}$ yields a higher *E*. Specifically, when the interlayer travel cost is high, OD pairs in the network tend to not choose the high-speed layer. As the transfer cost decreases (this change is reflected by the travel speed in the paper), more and more OD pairs begin to choose the high-speed layer, thereby improving the overall efficiency of the network. The behavior of *U* is more complex: as $v_{0}^{I}$ increases, the *U* improves significantly and the curves are accompanied by sharp fluctuations and jumps as γ increases. The variation of *J* is also closely tied to both $v_{0}^{I}$ and γ. A critical case occurs when $v_{0}^{I}=0.00001$. Here, *E* remains at its lowest value, *U* is 0. This confirms that without inter-layer transmission capacity, the advantages of the hierarchical network cannot be realized. The high-speed layer becomes isolated, and the system degenerates to operating solely on the low-speed layer.

## 4. Conclusions

This paper first constructs a two-layer transportation network based on node betweenness in a two-dimensional square lattice, and the concept of speed ratio is introduced to quantify the speed difference of the multilayer network. The multilayer network is integrated into the following model: the user equilibrium flow assignment strategy model based on the Bureau of Public Roads function. Subsequently, using network efficiency, high-speed layer utilization ratio, and proportion of congested edges as metrics, it examines the impacts of speed ratio between high-speed and low-speed layers, low-speed layer topology, and interlayer transport velocity on the model. Key findings include: (1) Increasing speed ratio γ elevates network efficiency, intensifies high-speed layer congestion while alleviating low-speed layer congestion, with high-speed layer utilization ratio showing nonlinear non-monotonic trends; (2) At low *Q*, the advantages of the high-speed layer can be fully realized; (3) As the number of nodes in the high-speed layer increases, this leads to a stronger response of *E* to γ, a higher final value of *E*, a more stable increase in *U*, and a significant suppression of *J*; (4) Long-range edges in ER random and BA scale-free networks improve efficiency, alleviating traffic congestion; (5) Introducing inter-layer travel speed may enhance efficiency in specific parameter regimes. These insights reveal the critical role of interlayer speed differences in network performance, offering theoretical and practical guidance for transportation planning. The multi-layer network construction algorithm for high-speed and low-speed layers proposed in this paper can be further applied to the testing and study of other transportation models. Research in this area can serve not only as a reference for planning new high-speed and low-speed multi-layer transportation networks, but also for optimizing and enhancing the performance of existing ones.

## Figures and Tables

**Figure A1:**
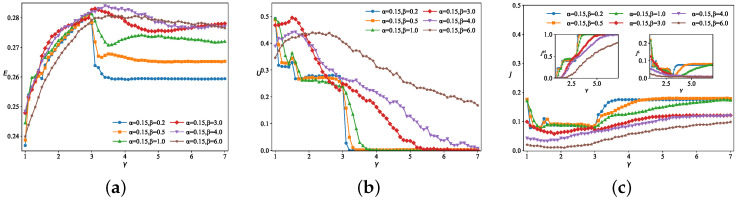
Effects of β on traffic dynamics for fixed α=0.15: (**a**) network efficiency *E*, (**b**) high-speed layer utilization ratio *U*, and (**c**) congestion factor *J* as functions of the inter-tier speed ratio γ.

**Figure A2:**
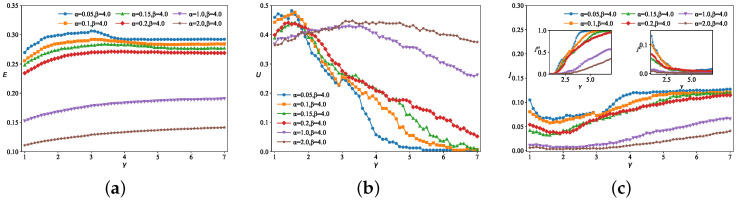
Effects of α on traffic dynamics for fixed β=4.0: (**a**) network efficiency *E*, (**b**) high-speed layer utilization ratio *U*, and (**c**) congestion factor *J* as functions of the inter-tier speed ratio γ.

**Figure A3:**
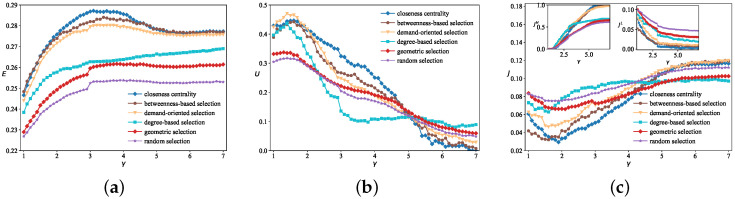
Comparison of different high-speed-layer node selection strategies. The curves depict (**a**) network efficiency *E*, (**b**) high-speed-layer utilization ratio *U*, and (**c**) congestion factor *J* as functions of the inter-tier speed ratio γ.

**Figure A4:**
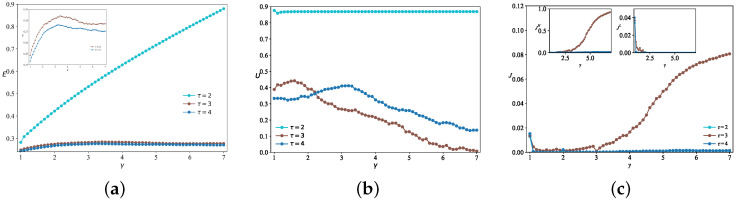
For different values of τ, the curves depict (**a**) network efficiency *E*, (**b**) high-speed-layer utilization ratio *U*, and (**c**) congestion factor *J* as functions of the inter-tier speed ratio γ.

**Figure A5:**
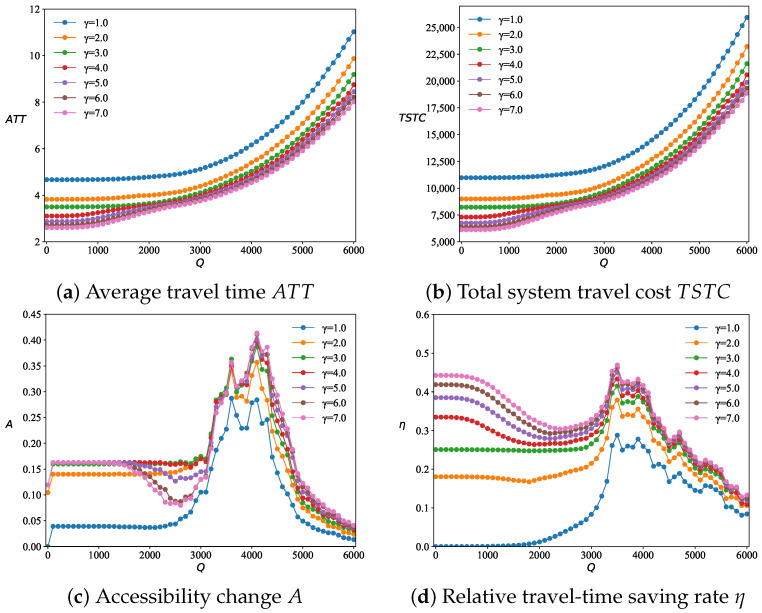
Under different values of γ, the four indicators—(**a**) the average travel time ATT, (**b**) the total system travel cost TSTC, (**c**) accessibility change *A*, and (**d**) the relative travel-time saving rate η—vary with traffic flow *Q*.

**Figure A6:**
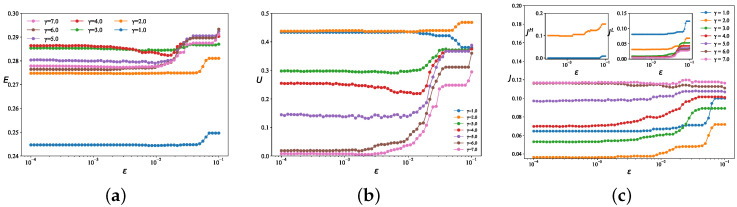
Numerical sensitivity of (**a**) network efficiency *E*, (**b**) high-speed layer utilization ratio *U*, and (**c**) congestion measures (*J*, $J^{H}$, and $J^{L}$) to the Frank–Wolfe stopping tolerance ε under Q=3500. This figure is used to assess convergence robustness rather than to infer transport-system behavior.

**Figure A7:**
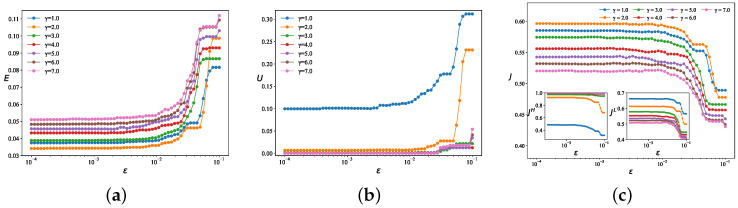
Numerical sensitivity of (**a**) network efficiency *E*, (**b**) high-speed layer utilization ratio *U*, and (**c**) congestion measures (*J*, $J^{H}$, and $J^{L}$) to the Frank–Wolfe stopping tolerance ε under Q=6000. This figure is used to assess convergence robustness rather than to infer transport-system behavior.

**Figure A8:**
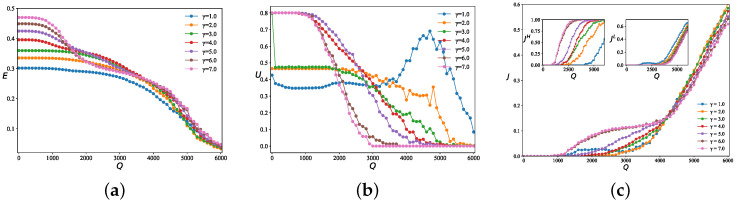
The curves depict (**a**) network efficiency *E*, (**b**) high-speed layer utilization ratio *U*, and (**c**) congestion factor *J* for different networks as functions of the traffic flow *Q* under different inter-tier speed ratios γ, with ε=0.001.

## Data Availability

The data that support the findings of this study are available upon reasonable request from the corresponding author.

## Appendix A

### Appendix A.1. Discussion of the α and β Parameters

With the lower layer fixed as a two-dimensional square lattice ($N^{L}=49$, $N^{H}=9$) and parameters τ=3, $t_{ij}^{I}=0$, Q=3500 and ε=0.01. In Equation (3), the parameters α and β determine the sensitivity of the link travel cost to the flow-to-capacity ratio. To examine their effects, we fix the two-dimensional square lattice network and vary β with α=0.15, and vary α with β=4.0, respectively. For fixed α=0.15, the results are shown in Figure A1. The efficiency *E* generally increases with γ when γ<3.0, whereas its subsequent behavior depends on β. When β is small, *E* decreases sharply after γ≈3. For all β, the utilization ratio *U* exhibits a non-monotonic dependence on γ. In particular, when β is small, *U* rapidly approaches zero after γ≈3; by contrast, when β is large, *U* first increases and then decreases as γ increases. When γ>3.0, the congestion factor *J* increases with γ.

For fixed β=4.0, the results are shown in Figure A2. Increasing α leads to a clear reduction in the network efficiency *E* over the whole range of γ. When γ>3.0, *U* increases with α. As for the congestion factor *J*, a larger α keeps *J* at a relatively low level over the entire range of γ.

Overall, the numerical results show that both α and β significantly affect the dependence of *E*, *U*, and *J* on the inter-tier speed ratio γ. A larger β generally helps maintain higher efficiency and lower congestion, whereas a larger α is usually associated with lower efficiency and weaker overall congestion.

### Appendix A.2. Discussion of High-Speed Layer Node Selection Strategy

With the lower layer fixed as a two-dimensional square lattice ($N^{L}=49$) and parameters τ=3, $t_{ij}^{I}=0$, Q=3500 and ε=0.01, we compare the betweenness-based selection strategy with several alternative node-selection schemes, including closeness-centrality-based selection, degree-based selection, random selection, geometric selection, and demand-oriented selection. Figure A3 shows the corresponding network efficiency *E*, high-speed-layer utilization ratio *U*, and congestion factor *J* as functions of the inter-tier speed ratio γ.

As shown in Figure A3a, when γ<3.0, the efficiency *E* increases with γ for all strategies. Among all the compared methods, the closeness-centrality-based strategy yields the highest *E* over most of the range of γ, while the proposed betweenness-based selection remains very close to it and is consistently superior to the other selection strategies. Figure A3b shows that, for all strategies, the utilization ratio *U* exhibits a peak around γ≈1.5. The closeness-centrality-based and betweenness-based strategies show relatively high utilization in the intermediate-γ regime, indicating that the high-speed layer is more frequently used under these two selection rules. When γ becomes large, the differences among the strategies gradually decrease, and all curves approach relatively low values. The overall congestion factor *J* in Figure A3c exhibits a non-monotonic pattern in the small-γ regime, followed by an overall increasing trend as γ continues to increase. In the low-γ regime, the proposed betweenness-based selection gives the smallest *J* among all the compared strategies. In the high-γ regime, the overall *J* under degree-based, random, and geometric selection is slightly lower than that under closeness-centrality-based and betweenness-based selection. However, the insets show that the congestion patterns differ between layers: $J^{H}$ generally increases with γ, whereas $J^{L}$ decreases with γ. In particular, at large γ, the betweenness-based and closeness-centrality-based strategies correspond to relatively small $J^{L}$ but comparatively large $J^{H}$, indicating that congestion is shifted more strongly to the high-speed layer. Overall, these results show that the proposed betweenness-based selection is not optimal for every metric and every value of γ, but it remains competitive across all three indicators. In particular, its efficiency is very close to that of the best-performing closeness-centrality-based strategy, while in the low-γ regime its overall congestion is lower than that of most of the other strategies. Since the high-speed layer in our model mainly serves to intercept major cross-network flows and relieve bottlenecks, betweenness centrality provides a node-selection criterion that is closely related to this function. In this sense, it is consistent with the intended role of the high-speed layer as a backbone supported by key bridging nodes.

### Appendix A.3. Discussion of the Distance Parameter τ in the High-Speed Layer

In the proposed construction algorithm, τ plays a dual role. During node selection, once a node is added to the high-speed layer, candidate nodes within a topological distance smaller than τ are removed, thereby enforcing a minimum spacing among high-speed nodes. During edge construction, high-speed links are created between node pairs whose distance is exactly τ. Therefore, τ directly controls both the spacing of high-speed nodes and the characteristic reach of high-speed edges. This parameter is introduced to reflect the structural feature that real high-speed facilities are usually sparser and more long-ranged than the pre-existing low-speed layer. Without such a spacing constraint, the upper layer could become unrealistically dense, thereby losing the intended hierarchical distinction between the two layers.

In this set of experiments, we fix the number of nodes and edges in the low-speed layer and vary only τ=2,3,4, generating the high-speed layer according to the algorithm “until no more eligible nodes remain”. Therefore, changing τ not only alters the spacing between high-speed nodes but also simultaneously affects the number of feasible nodes in the high-speed layer and its connection structure. As shown in Figure A4, τ has a significant impact on network performance. First, in terms of efficiency *E*, for τ=2, *E* increases notably with the speed ratio γ; whereas for τ=3 and τ=4, *E* only improves slightly over a small range of γ and then reaches a plateau. This indicates that a smaller τ leads to a denser high-speed layer, which provides more access points and more usable shortcuts, thereby facilitating the translation of speed advantages into global efficiency gains. Conversely, when τ is too large, although the high-speed edges have longer spans, the limited coverage of the high-speed layer and reduced access opportunities significantly constrain the improvement in efficiency. Second, regarding the utilization ratio *U* of the high-speed layer, *U* remains consistently high for τ=2, while for τ=3 and τ=4, *U* is markedly lower and exhibits a declining trend over a wide range of γ. This suggests that when the high-speed layer is too sparse, even a further increase in the upper-layer speed can hardly be stably exploited by most OD pairs. Instead, traffic tends to concentrate on a few high-speed edges, and under the effect of congestion feedback, some paths revert to the low-speed layer, leading to a decrease in *U*. Finally, concerning the congestion index *J*, for τ=2, both total and layer-wise congestion are close to zero, whereas for τ=3 and τ=4, the total congestion *J* and the high-speed layer congestion $J^{H}$ increase significantly with γ. This indicates that under larger τ, the high-speed layer has fewer nodes and edges, and the speed advantage attracts traffic to concentrate on the limited high-speed routes, making it easier for bottlenecks to form in the upper layer. At the same time, the low-speed layer congestion $J^{L}$ remains at a low level, implying that increasing γ still induces a transfer of congestion from the low-speed layer to the high-speed layer. Overall, τ significantly influences the coverage and bottleneck distribution of the high-speed layer, thereby altering the magnitude of efficiency improvements and congestion shifts. Nevertheless, within the range τ=2,3,4, the core conclusion of this paper remains consistent: increasing the inter-layer speed ratio γ is generally beneficial for improving network efficiency, but it also attracts more traffic to the high-speed layer and induces more pronounced upper-layer congestion when the high-speed layer is relatively sparse.

### Appendix A.4. Supplementary Analysis of Multiple Transport Performance Measures

To evaluate the network performance under different scenarios, four indicators are adopted in this study, namely the average travel time (ATT), total system travel cost (TSTC), accessibility change under a travel-time threshold (*A*), and the relative travel-time saving rate (η). The average travel time is defined as

(A1) $$ATT=\frac{1}{N^{L}\left(N^{L}-1\right)}\sum_{i\neq j}t_{ij},$$

where $N^{L}$ denotes the total number of nodes in the low-speed-layer network; $t_{ij}$ denotes the travel time from node *i* to node *j*. The average travel time (ATT) represents the average level of travel time among all origin–destination node pairs in the low-speed-layer network, that is, the average travel efficiency of the network. From an overall perspective, it describes how long it takes, on average, to travel between any two nodes in the network. A smaller ATT indicates a shorter average travel time, better overall network accessibility, and higher travel efficiency. In contrast, a larger ATT indicates a longer average travel time and lower overall travel efficiency. The total system travel cost is defined as

(A2) $$TSTC=\sum_{i\neq j}t_{ij},$$

where $t_{ij}$ denotes the travel time from node *i* to node *j*. The total system travel cost (TSTC) represents the sum of travel times over all node pairs. It reflects the total time cost borne by the entire system. In other words, it describes the total amount of travel time required for the whole network to complete all origin–destination connections. A smaller TSTC indicates a lower total travel cost and a better overall operating condition of the network, whereas a larger TSTC indicates a greater accumulation of travel time and a heavier system-wide burden. The accessibility change under a given travel-time threshold is defined as

(A3) $$A=\frac{1}{N^{L}\left(N^{L}-1\right)}\sum_{i\neq j}\left[\delta \left(t_{ij}^{\mathrm{with}}\leq t_{c}\right)-\delta \left(t_{ij}^{\mathrm{without}}\leq t_{c}\right)\right],$$

where $N^{L}$ denotes the total number of nodes in the low-speed-layer network; $t_{ij}$ denotes the travel time from node *i* to node *j*; $t_{ij}^{\mathrm{with}}$ and $t_{ij}^{\mathrm{without}}$ denote the travel times from node *i* to node *j* in the scenarios with and without the high-speed layer, respectively. The travel-time threshold $t_{c}$ is defined as $t_{c}=1.5\;{\bar{t}}^{\mathrm{without}}$. ${\bar{t}}^{\mathrm{without}}$ is the average travel time in the scenario without the high-speed layer. The term δ(·) is an indicator function, which takes the value 1 if the travel time from node *i* to node *j* does not exceed $t_{c}$, and 0 otherwise. The accessibility change (*A*) measures the change in the proportion of node pairs that can be reached within a given travel-time threshold $t_{c}$ when the high-speed layer is introduced, compared with the case without the high-speed layer. If A>0, the number of node pairs reachable within the prescribed time threshold increases, indicating an improvement in accessibility. If A=0, the accessibility within the threshold remains essentially unchanged. If A<0, the number of node pairs reachable within the threshold decreases, indicating a decline in accessibility. Therefore, a larger *A* indicates a more significant contribution of the high-speed layer to improving threshold-based accessibility. In essence, this indicator describes the extent to which the high-speed layer improves network accessibility in terms of whether destinations can be reached within a limited time budget. The relative travel-time saving rate is defined as

(A4) $$\eta =\frac{\sum_{i\neq j}\left(t_{ij}^{\mathrm{without}}-t_{ij}^{\mathrm{with}}\right)}{\sum_{i\neq j}t_{ij}^{\mathrm{without}}},$$

where $t_{ij}^{\mathrm{with}}$ and $t_{ij}^{\mathrm{without}}$ denote the travel times from node *i* to node *j* in the scenarios with and without the high-speed layer, respectively. The relative travel-time saving rate (η) represents the proportion of total travel time saved after the introduction of the high-speed layer, relative to the scenario without the high-speed layer. It reflects the overall time-saving effect brought by the high-speed layer. If η>0, the total travel time is reduced after the high-speed layer is introduced, indicating that the high-speed layer generates travel-time savings. If η=0, the total travel time remains nearly unchanged, indicating no obvious improvement. If η<0, the total travel time increases after the introduction of the high-speed layer, suggesting that the high-speed layer fails to produce a positive effect and may even reduce network efficiency. Thus, a larger η indicates a stronger time-saving effect, whereas a smaller η indicates a weaker contribution of the high-speed layer to travel-time reduction.

As shown in Figure A5, with the lower layer fixed as a two-dimensional square lattice $\left(N^{L}=49,N^{H}=9\right)$ and the parameters set to τ=3, $t_{ij}^{I}=0$, Q=3500, and ε=0.01, (a) ATT increases monotonically with *Q*, but decreases consistently as γ increases for any given *Q*, showing that a larger inter-tier speed ratio reduces the average travel burden; (b) TSTC rises rapidly with *Q*, yet for any fixed demand level it decreases as γ increases, confirming that the speed advantage of the high-speed layer can effectively reduce the overall system travel cost; (c) the accessibility change *A* is positive for nearly all values of γ, indicating that the high-speed layer generally improves threshold-based accessibility, with the strongest gains appearing under intermediate-to-high traffic demand and weakening at very high demand; (d) the relative travel-time saving rate η remains positive when γ>1 and generally becomes larger as γ increases, with a pronounced peak at intermediate demand, implying that the time-saving benefit of the high-speed layer is greatest under moderate traffic load.

### Appendix A.5. Discussion of the Convergence Threshold ε

The convergence threshold ε in the Frank–Wolfe algorithm is a numerical stopping tolerance, rather than a physical or behavioral attribute of the multilayer transport system. Therefore, variations in the model outputs with respect to ε should be interpreted as indicators of algorithmic sensitivity and convergence quality, not as substantive transport phenomena. We use this section only to verify the numerical robustness of the reported results.

As shown in Figure A6, when the stopping tolerance ε is sufficiently small, the values of *E*, *U*, *J*, $J^{H}$, and $J^{L}$ remain nearly unchanged for each γ, indicating that the Frank–Wolfe iterations have effectively converged. In this regime, the differences among curves mainly reflect the model structure rather than the numerical stopping rule. As ε increases, visible deviations from these plateau values begin to appear, especially in the utilization and congestion-related measures. These changes should be interpreted as symptoms of premature termination or incomplete convergence, rather than as substantive transport-system responses to ε.

With the low-speed layer configured as a two-dimensional square lattice (lower-layer node count $N^{L}=49$, upper-layer node count $N^{H}=9$), and under the conditions of traffic demand Q=6000, τ=3, and $t_{ij}^{I}=0$, Figure A7 presents the numerical sensitivity test for the higher-demand case. Compared with Figure A6, the results are more sensitive to the stopping tolerance, indicating that convergence control becomes more important in the high-demand regime. When ε is sufficiently small, all metrics remain stable, supporting the numerical reliability of the corresponding solutions. However, for larger ε, the deviations become more pronounced, suggesting that the algorithm may terminate before reaching an adequately converged user-equilibrium solution. Therefore, the ε-dependent patterns are treated only as evidence of numerical sensitivity, not as behavioral or planning implications.

Overall, the sensitivity analysis confirms that ε must be chosen sufficiently small before drawing substantive conclusions from the model. Accordingly, all behavioral and planning interpretations in this paper are based only on the converged regime.

## Appendix B

### Appendix B.1. Inter-Tier Speed Ratio Effect on Network at ε = 0.001

Fixing τ=3, $N^{L}=49$, $N^{H}=9$, $t_{ij}^{I}=0$, and ε=0.001, and averaging over 50 independent simulation runs, we investigate the impact of the inter-tier speed ratio γ and traffic flows *Q* on efficiency *E*, high-speed layer utilization *U* and multilayer network congestion factor *J*. Figure A8a shows that all curves start with a slow decline from Q=0, and larger values of γ correspond to higher initial values. Although the declining trends are relatively smooth, the efficiency curves for different γ tend to converge under high-flow conditions. Figure A8b illustrates that a larger γ results in higher utilization *U* under low-flow conditions, but lower *U* under high-flow conditions. At low loads, the high-speed layer is highly attractive; at high loads, however, its limited capacity causes traffic to be offloaded or rerouted to low-speed layers, leading to a sharp decline in utilization. The case of γ=1.0 is the only one where a peak in *U* is observed, indicating that when the speeds of different layers are identical, the utilization of the high-speed layer is optimized under high-flow conditions, while excessively high flow leads to a degradation in utilization. Figure A8c shows that as the traffic demand *Q* increases from 0 to 6000, the congestion factor of the low-speed layer $J^{L}$ exhibits a clear monotonic upward trend for all values of γ, with smaller γ corresponding to earlier and higher increases in $J^{L}$. The overall network congestion factor *J* remains close to zero under low to medium demand, then rises with increasing *Q*, showing a generally monotonic increase. As for the congestion factor of the high-speed layer $J^{H}$, when γ is small, $J^{H}$ remains at a very low level throughout nearly the entire range. As γ increases, $J^{H}$ rises significantly and reaches relatively high values under medium to high demand, with the curve initially increasing rapidly and then tending to saturate. This indicates that larger γ causes traffic to concentrate on the high-speed layer earlier and more intensely, leading to earlier visible congestion in the high-speed layer.

### Appendix B.2. The Influence of the Inter-Tier Speed Ratio on Network for Different Traffic Demand

With the lower layer fixed as a two-dimensional square lattice ($N^{L}=49$, $N^{H}=9$) and parameters τ=3, $t_{ij}^{I}=0$ and ε=0.01, Figure A9 illustrates the system behavior as the total traffic demand *Q* increases. The overall efficiency *E* decreases consistently with growing *Q*. Utilization *U* rises under low demand, exhibits a peak-and-decline pattern under moderate demand, and trends downward under high demand. As γ increases, congestion in the high-speed layer becomes more severe, while congestion in the low-speed layer is alleviated.
